# Leveraging AI to Advance Age-Friendly Care in the Veterans Health Administration

**DOI:** 10.2196/75686

**Published:** 2026-02-23

**Authors:** Elizabeth Fine Smilovich, Megha Kalsy, Kimberly Wozneak, Quratulain Syed, Laurence M Solberg

**Affiliations:** 1 Geriatrics Research, Education, and Clinical Center (GRECC), Veterans Affairs Northeast Ohio Healthcare System, 10701 East Boulevard, Cleveland, OH, 44106, United States, 1 2167913800 ext 66362; 2 Frances Payne Bolton School of Nursing, Case Western Reserve University, Cleveland, OH, United States; 3 College of Public Health and Health Professions, University of Florida, Gainesville, FL, United States; 4 Geriatrics and Extended Care, Veterans Health Administration, Washington, DC, United States; 5 Geriatric Research Education and Clinical Center (GRECC), Atlanta VA Medical Center, Atlanta, GA, United States; 6 Geriatrics Research, Education, and Clinical Center (GRECC), NF/SG VHS, Malcom Randall VA Medical Center, Gainesville, FL, United States

**Keywords:** age-friendly health systems, 4Ms framework, aging, artificial intelligence, technology, older adults, geriatrics, Veterans Health Administration

## Abstract

The aging population presents a pressing challenge for health care systems, necessitating effective strategies to address the complex needs of older adults. The Veterans Health Administration in the Department of Veterans Affairs (VA), the largest integrated health care system in the United States, has embraced the Age-Friendly Health Systems (AFHS) initiative from the Institute for Healthcare Improvement to ensure safe and high-quality care for older veterans. In AFHS, health care providers consistently use the evidence-based 4Ms framework (what matters, medication, mentation, and mobility) to deliver comprehensive care for older adults in all care settings.

This viewpoint paper explores the potential of artificial intelligence (AI) to enhance the evidence-based implementation of the AFHS 4Ms framework in the VA to provide optimal care for older adults. By leveraging AI technologies, such as natural language processing, machine learning, large language models, clinical decision support, and data analytics, this viewpoint examines the opportunities and challenges of using AI to support the 4Ms domains in a large, integrated health care system. Furthermore, it discusses the potential benefits of integrating AI-driven decision support systems and predictive analytics to personalize care, reduce polypharmacy and potentially inappropriate medications, enhance cognitive and mood assessments, and better identify mobility issues and interventions. By examining the intersection of AI and age-friendly care in the VA, this viewpoint highlights the transformative potential of AI to expand 4Ms care and improve the experience of providers and older adults across diverse health care settings.

## Introduction

The United States is experiencing a significant demographic shift, characterized by a rapidly aging population. By 2050, an estimated 82 million individuals will be aged 65 years or older [1]. The Veterans Health Administration in the Department of Veterans Affairs (VA) is the largest integrated health care system in the United States, and a significant number of older veterans are enrolled in health care provided by the VA [2]. An estimated 30% of adults aged 65 years or older could benefit from specialized care from geriatricians; however, there continues to be a scarcity of trained geriatricians despite innovative solutions to meet the rising demands within the geriatric workforce [3]. To meet the increasingly complex care needs of the growing older veteran population, the VA joined the Age-Friendly Health Systems (AFHS) movement in March 2020 [4]. The AFHS initiative within the VA continues to build on decades of evidence-based care and innovation in geriatrics, which aligns with the 4Ms framework (what matters, medication, mentation, and mobility) [5] and supports the VA’s goal of becoming a high reliability organization [4,6-8].

As efforts to train health care professionals to optimize a geriatric-friendly workforce continue, artificial intelligence (AI) can be leveraged to supplement these efforts and support the geriatric workforce in providing high-quality care to older adults with multiple chronic conditions.

## The VA as an Age-Friendly Health System

The AFHS framework emphasizes developing and aligning care plans for older adults to meet the goals that matter most to them. AFHS was established in 2017 by the John A. Hartford Foundation and the Institute for Healthcare Improvement (IHI) in collaboration with the American Hospital Association and the Catholic Health Association of the United States. This initiative promotes a comprehensive framework known as the 4Ms, which focuses care on the “what matters” element to the patient and on assessing medication, mentation, and mobility to ensure evidence-based and reliable care for older adults [7,8].

The VA has made important strides in adopting the 4Ms on an enterprise scale. As of July 2025, the VA has implemented AFHS in 455 clinical sites, with all sites receiving Level 1 Participation in AFHS recognition from the IHI [9]. Level 1 recognition means that the clinical site has described how the 4Ms will be put into practice. Of these sites, 297 have achieved Level 2 Committed to Care Excellence recognition from the IHI. Level 2 recognition means that the clinical site has tracked and shared the number of older adults reached with the 4Ms over at least 3 months and has described how the 4Ms will be sustained in practice. The implementation and spread of AFHS in the VA has reached 164 Veteran Affairs Medical Centers nationwide.

The central focus of the AFHS movement is to prioritize “what matters” to older adults—the first “M”—placing their specific health outcome goals and care preferences at the forefront across all care settings. The VA has developed several ways, through different programs, to elicit this information, such as the Whole Health Mission, Aspiration, Purpose inquiry; the Patient Priorities Care survey; and the Well-Being Signs tool. These tools are accessible through an age-friendly 4Ms template and can be viewed, if previously populated, when the template is opened. Health care providers looking to readily view this information to position the visit in what matters to the patient must open the template. Currently, no function exists to easily elicit from the chart what a veteran’s health priorities may be if the template wasn’t previously used.

The second “M”—”what medication”—emphasizes limiting the use of high-risk medications and dose adjustments when a high-risk medication is essential to achieve what matters to older adults. Within the VA, there is active groundwork to support safer medication use: VIONE (Vital, Important, Optional, Not indicated), a nationwide deprescribing program with dashboards and risk tools embedded in the electronic health record (EHR), has been disseminated across more than 130 medical centers and supports pharmacist-led deprescribing [10]. VA informatics efforts have also applied natural language processing (NLP) to prescribing problems and are advancing AI- and machine learning (ML)-enabled decision support as part of a learning health system strategy [11]. Software built into the EHR notifies providers of medication interactions; however, currently, systems to review medications using Beer’s criteria (which identify medications potentially inappropriate for older adults) or to alert health care providers to medication-disease interactions (eg, anticholinergic medications in patients with cognitive impairment) are not standard and require the expertise of geriatric-trained providers and pharmacists.

The third “M”—“what mentation”—concentrates on preventing, identifying, treating, and managing conditions such as dementia, depression, and delirium across care settings. Current use of the AFHS template with embedded cognitive and mood screening tools has improved access to these evidence-based assessments. Implementation, although progressing, faces challenges related to time and documentation constraints. Although there is a program for mental health clinicians to initiate and review mental health assessments (Mental Health Assistant), there is no repository within the EHR to easily view past screening results and actions; the AFHS template must be opened. Unless a veteran presents for cognitive evaluation to a mental health or geriatric specialist, the current system relies on individual providers to determine the best time to screen for cognitive impairment, often after warning signs for dementia are exhibited. Electronic frailty indices, based on predictive models such as the VA Frailty Index and the Care Assessment Needs score, exist within the VA system [12]; however, they are not used in clinical settings to identify patients at risk of cognitive decline.

The final “M”—“what mobility”—promotes mobility evaluation to encourage older adults to safely engage in daily physical activity to maintain function and pursue what matters to them. Current use of the AFHS template using embedded mobility screening tools prompts health care providers to perform either a history or physical examination-based screen. Real-time remote monitoring of gait or fall risk for ambulatory care patients is not yet part of routine care, and other potential predictors, such as the Care Assessment Needs score, do not correlate well with physical functioning [13].

Individually, each “M” has the potential to improve health outcomes, reduce low-quality services, and increase the use of cost-effective interventions. Introducing the 4Ms approach as part of AFHS improves care for older veterans [4]. To promote the AFHS 4Ms approach and offer better care to the older population, the VA must leverage all tools, including the potentially transformative use of appropriate AI techniques.

AI is a rapidly developing technology that encompasses various fields of knowledge and uses a wide range of techniques. Its primary goal is to simulate and extend human intelligence through machines [14]. Within the field of AI, research areas include expert systems, ML, robotics, decision support systems, and pattern recognition. There are many potential applications of AI in health care for older adults, such as clinical decision support (CDS) systems, robotics for physical rehab and physiotherapy exercises, NLP and speech analysis, medical imaging and video processing, wearable devices and signal processing from digital watches that track health data, and using ML for targeted drug therapy [15,16].

AI has already demonstrated its potential in geriatric care by addressing challenges such as caregiver shortages and resource disparities. Researchers increasingly believe that AI may further enhance the well-being of older adults by redistributing health care resources, including nursing and other professional support. Numerous studies have explored the application of AI in geriatric care, focusing on areas such as Alzheimer disease care, disease recognition, and medication reminders [17].

Our viewpoint is that appropriate use of AI could better use and integrate the 4Ms across the VA. The implementation and sustainment of 4Ms care require scalable and efficient strategies to deliver equitable, evidence-based care to reach all older adults. We propose leveraging AI technologies, such as NLP, ML, ambient AI, CDS, and data analytics to support the AFHS movement. This manuscript delves into the opportunities and challenges in using AI to support the 4Ms domains within the VA system.

## Use of AI for 4M-Based Care at the VA

**Figure 1.**
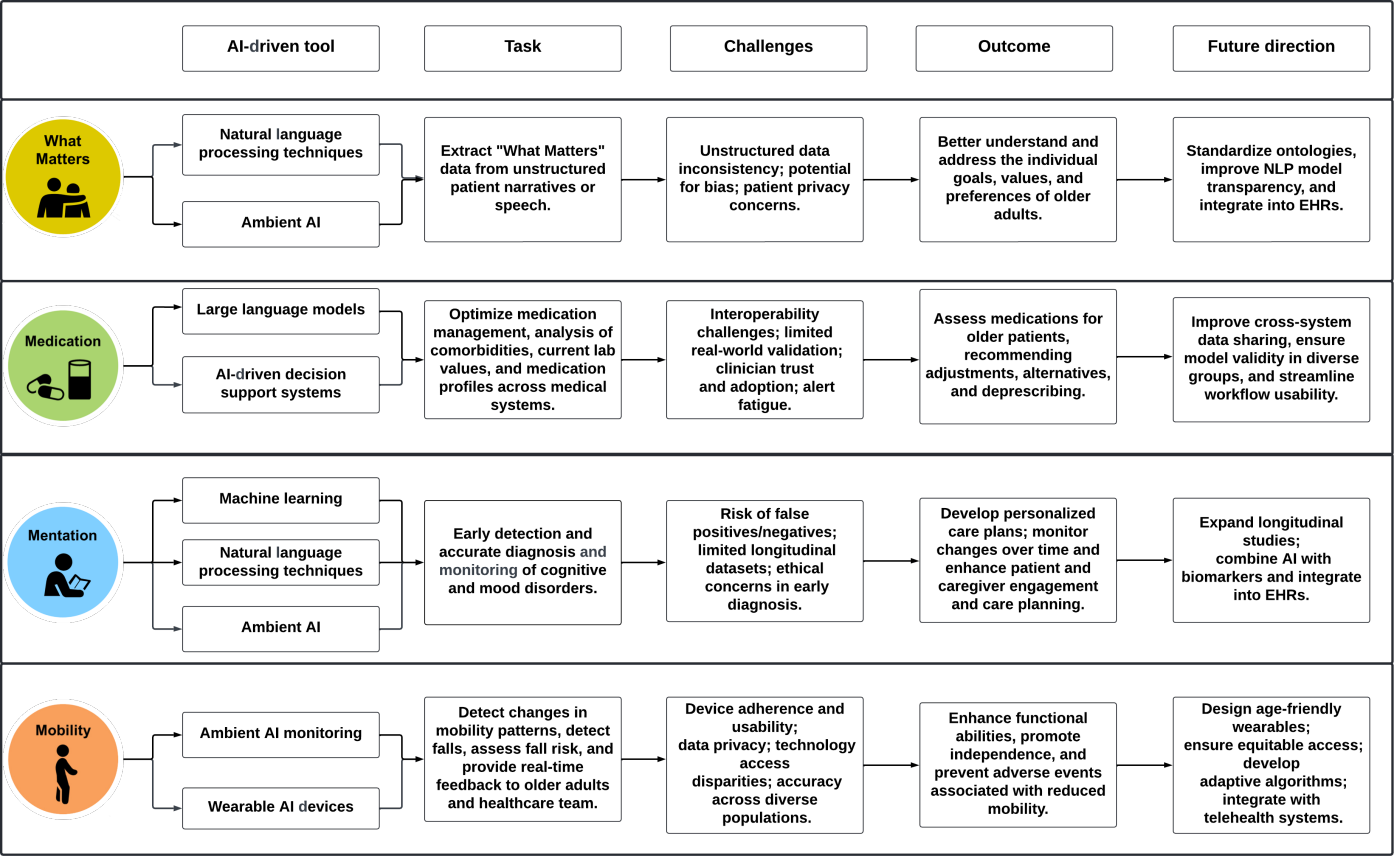
Proposed use of artificial intelligence (AI) for age-friendly 4Ms-based care. EHR: electronic health record; NLP: natural language processing.

Although AI is rapidly advancing, its application to AFHS and the 4Ms framework has not been systematically characterized. To date, no research has comprehensively assessed the scope, trends, effectiveness, or gaps in AI-driven approaches to AFHS within the VA. This lack of synthesis represents a barrier to advancing implementation and scaling of age-friendly care. By leveraging AI methods such as NLP, ML, predictive modeling, and advanced data analytics, with emerging opportunities in deep learning and large language models (LLMs), there is significant potential to transform large volumes of unstructured and structured clinical data into actionable insights that enhance evidence-based, person-centered care for older veterans within the 4Ms framework. Figure 1 describes the proposed use of AI for age-friendly 4Ms-based care.

### What Matters

Using AI in health care for older adults can expand the available tools to improve care delivery and assist health care professionals in personalizing medicine for this population. Implementing AI within the context of AFHS, with a focus on what matters to the older adult, may enable more efficient development of care plans that are both personalized and optimized. The AFHS 4Ms framework represents a paradigm shift in the care of older adults by emphasizing alignment of clinical practice guidelines with patients’ health goals and lifestyle preferences.

Extraction and compilation of EHR data across health care systems regarding patients’ priorities and goals remain challenging. AI tools such as NLP and ML can support clinical decision-making by identifying and synthesizing these priorities, thereby assisting clinical teams in formulating care plans centered on what matters to older adults. NLP, a branch of AI, enables computers to understand, extract, and analyze information from human language in clinical text and documentation. These techniques can extract insights from unstructured patient narratives, including documentation of goals, life-sustaining treatment, and advance care planning, to provide clinicians with a clearer understanding of patients’ values and preferences.

Recent work in this area has demonstrated promising applications. For example, Gleason et al [18] developed a rule-based NLP system to classify patient portal messages from older adults by the 4Ms domains (including the “what matters” domain) and achieved an area under the curve of 0.90 in identifying messages sent by care partners [18]. Similarly, Min et al [19] developed an NLP algorithm to automatically capture daily care experience preferences from narrative data, demonstrating how AI can operationalize the “what matters” domain at scale [19].

Beyond text-based extraction, emerging technologies such as ambient AI (ambient scribing) can capture and synthesize clinical discussions in real time, including conversations around “what matters,” to further support AFHS [20]. Integrating these AI-enabled insights into clinical workflows can promote more patient-centered care, facilitate shared decision-making, and improve patient satisfaction.

While some scholars caution that reliance on AI-driven clinical decision algorithms could risk a return to paternalistic models of care, AFHS emphasis on the “what matters” domain provides an important safeguard. By ensuring that AI tools are designed and implemented with patient priorities at the center, AFHS can help expand personalized medicine for older adults in a manner that enhances, rather than replaces, the patient voice.

### Medications

In the “medication” domain, AI-enabled CDS can help optimize pharmacotherapy for older adults by screening for high-risk prescribing, drug-drug interactions, renal-dose mismatches, and deprescribing opportunities at the point of care. Contemporary reviews show that ML models can predict adverse drug events from EHR data and that CDS interventions in older populations improve medication safety and deprescribing outcomes [21]. Emerging LLM applications extend this capability. LLMs are advanced AI systems trained on large-scale text data to understand and generate human language. The term “large” refers to the size of the datasets that assist in learning patterns in language. The term “language” reflects the model’s ability to understand, summarize, translate, and code text. The term “model” refers to the computational system that learns patterns in data to generate predictions or output. A 2025 scoping review mapped how generative AI and LLMs mitigate medication-related harm across the medication-use process [22] and a cohort study published in *JMIR Aging* demonstrates that an LLM-based pipeline can identify deprescribing opportunities for older adults, while also noting limits in complex clinical scenarios [23].

Modernization of medication reconciliation workflows highlights progress and remaining integration gaps that AI-driven CDS can address. These developments indicate that AI, including NLP, ML, and LLMs, may be potentially used to augment medication safety across the AFHS framework by surfacing risks, proposing safer alternatives and dose adjustments, and flagging candidates for deprescribing—particularly when embedded in workflows and paired with pharmacist and clinician review.

### Mentation

AI also holds promise in the “mentation” domain, where timely identification and management of cognitive and mood disorders are critical. ML approaches trained on large datasets can aid early detection and differential diagnosis of cognitive impairment (eg, Alzheimer disease and mild cognitive impairment). NLP applied to clinical notes improves delirium ascertainment that is often undercoded in structured data [24]. In addition, prototype ambient and patient-facing systems such as the SeVA prototype system use artificial emotional intelligence with chatbot-based monitoring and 2 mobile apps to provide timely checks and health status recording; early deployments report feasibility for detecting delirium and supporting nursing workflows [25]. Similarly, a recent study demonstrated the feasibility of using ML to analyze Wi-Fi–based motion sensor data to predict depression among older adults [26]. Together, these AI methods can synthesize cognitive testing, narrative documentation, and real-time signals to inform personalized care plans and track changes over time.

### Mobility

AI can strengthen the “mobility” domain by continuously detecting changes in gait and activity, assessing fall risk, and triggering timely interventions. Ambient and wearable sensing, ranging from step counters and actigraphy to contactless in-home systems, can monitor mobility patterns and surface deviations that matter clinically. In real-world living settings for older people, an AI video–based fall detection system reduced time on the ground and accelerated staff response after unwitnessed falls, demonstrating an actionable feedback loop to care teams [27].

In-home smart apartment platforms have similarly generated automated health alerts from passive sensors to notify clinicians of mobility and health changes, supporting earlier evaluation and intervention [28]. Contactless radio frequency sensing has enabled continuous home-based monitoring of gait speed and impairment (eg, in Parkinson disease), offering a path to track functional decline between visits [29].

Complementary evidence from reviews shows that ambient assisted-living and remote passive-sensing technologies can detect falls, changes in activities of daily living, and health status shifts, informing prevention and individualized mobility care plans [30].

## The 4Ms as an Integrated Set

We propose implementing an integrated AI approach to the 4Ms. A prompted LLM extraction service will harvest 4Ms signals from structured and free-text EHR sources and patient messages (“what matters” domain preferences, high-risk medications, cognitive or behavioral changes, and falls or balance changes), building on evidence that LLMs can detect postoperative falls from clinical narratives [31]. A VA chat generative pretrained transformer chart reviewer will then compile the 4Ms into a provenance-linked 4Ms summary card, using structured data fields where permissible, and surface care gaps—such as missing goals-of-care documentation, high-risk prescribing, delirium language, or gait decline—as team-routable alerts. To ease documentation burden, ambient AI–assisted drafting and smart templates will prepopulate 4Ms documentation, supported by a previsit 4Ms gaps checklist and 2-click insertion into EHR notes. Success metrics could include entity-level *F*_1_-scores of at least 0.80 for key 4Ms concepts, note-level recall of at least 0.90 for any 4Ms signal, a 25% or greater reduction in documentation time, a 20% or greater increase in 4Ms completeness, improved usability scores, and demonstrable gains in goal-concordant care and time to action on fall risk and high-risk medications.

## Barriers and Challenges to Integrating AI Into AFHS and EHRs

There are numerous challenges to integrating AI into health care for older adults. One consideration is the potential degradation of face-to-face human contact with clinicians and health care professionals resulting from the implementation of these technologies. This could lead to fewer routine caregiver interactions and increased social isolation [15]. Another challenge, as previous studies have demonstrated, is that wearable devices in long-term care environments can be uncomfortable and the sensors may disturb sleep [32]. Considerations such as these must be at the forefront of discussions regarding AI implementation in the care of older adults.

While AI has the potential to support health care professionals in the provision of high-quality health care services for older adults, numerous concerns have been raised in research studies, including bias in AI algorithms, which have the potential to precipitate existing disparities in access to health care resources, and concerns about patient privacy in the setting of possible cybersecurity threats [15].

Implementing AI within the VA to support the evidence-based AFHS 4Ms framework presents multiple challenges and barriers. One major challenge is integrating AI with existing EHRs. EHRs are often complex and fragmented across various platforms, necessitating sophisticated data harmonization strategies to enable AI-driven insights without compromising data integrity [33]. Furthermore, ethical considerations are paramount, as AI systems must ensure patient privacy and confidentiality, consistent with HIPAA (Health Insurance Portability and Accountability Act) regulations, while also being transparent in decision-making processes to gain stakeholders’ trust [34]. The health system culture may pose another barrier, as clinicians may resist adopting AI tools due to perceived threats to clinical autonomy, potential disruptions to established workflows, and the learning curve associated with new technologies [35]. Addressing these issues requires a comprehensive strategy that aligns technological advancements with ethical standards and cultural readiness, involving stakeholders at all levels of the VA.

While the integration of AI into AFHS care within the VA presents exciting opportunities that may improve patient experience and allow older people to age in place and live in their homes longer, and decrease cognitive burden on clinicians and health care professionals, several challenges must be addressed. These include ensuring data privacy and security, data harmonization within the EHR, addressing potential biases in AI algorithms, and fostering user acceptance and trust in AI-powered systems. Overcoming these challenges requires interdisciplinary collaboration among health care professionals, data scientists, engineers, and policymakers supporting the AFHS to provide optimal personalized health care to older adults.

By applying a scientific lens to the intersection of AI and age-friendly care, this viewpoint paper has explored the transformative potential of AI in enhancing evidence-based implementation of the 4Ms framework. It highlights the opportunities and challenges in leveraging AI technologies to personalize care, improve medication management, enhance cognitive and mood assessments, and optimize mobility interventions for older adults across diverse health care settings. The findings of this study have implications for health care policymakers, administrators, and researchers seeking to maximize the benefits of AI in delivering high-quality, person-centered care for older adults.

## Conclusion

This viewpoint paper addresses the urgent need to develop effective strategies for the use of AI to enhance evidence-based implementation of the AFHS framework within the VA as a model for AI integration in a large, integrated health care system. AI tools such as NLP, ML, and data analytics can be leveraged to support effective implementation of 4Ms-based care across the VA health care system. Integrating AI-driven decision support systems and predictive analytics can personalize care, reduce polypharmacy, improve cognitive and mood assessments, and identify mobility issues more effectively. This viewpoint highlights the transformative potential of AI in expanding care for older adults by creating easier and more effective ways to implement 4Ms care, potentially improving outcomes and experience for older adults across the VA. Embracing AI in AFHS holds promise for enhancing care quality, optimizing resources, and improving well-being. Further research and collaboration are needed to fully harness AI’s potential in age-friendly care, leading to effective and person-centered health care solutions globally.

## Abbreviations

AFHS: Age-Friendly Health Systems
AI: artificial intelligence
CDS: clinical decision support
EHR: electronic health record
HIPAA: Health Insurance Portability and Accountability Act
IHI: Institute for Healthcare Improvement
LLM: large language model
ML: machine learning
NLP: natural language processing
VA: Department of Veterans Affairs
